# Iterative development of Stand Up Australia: a multi-component intervention to reduce workplace sitting

**DOI:** 10.1186/1479-5868-11-21

**Published:** 2014-02-21

**Authors:** Maike Neuhaus, Genevieve N Healy, Brianna S Fjeldsoe, Sheleigh Lawler, Neville Owen, David W Dunstan, Anthony D LaMontagne, Elizabeth G Eakin

**Affiliations:** 1 The University of Queensland, School of Population Health, Cancer Prevention Research Centre, Herston, Queensland, Australia; 2 Baker IDI Heart and Diabetes Institute, Melbourne, Victoria, Australia; 3 Monash University, Medicine, Melbourne, Australia; 4 The McCaughey Centre, School of Population Health, University of Melbourne, Melbourne, Australia; 5 The University of Western Australia, School of Sports Science, Exercise and Health, Perth, Australia; 6 The University of Melbourne, School of Population & Global Health, Melbourne, Australia

**Keywords:** Intervention development, Sedentary behaviour, Sitting time, Sit-stand, Physical activity, Postural transitions, Workplace, Workplace intervention, Office workers, Height-adjustable workstations

## Abstract

**Background:**

Sitting, particularly in prolonged, unbroken bouts, is widespread within the office workplace, yet few interventions have addressed this newly-identified health risk behaviour. This paper describes the iterative development process and resulting intervention procedures for the *Stand Up Australia* research program focusing on a multi-component workplace intervention to reduce sitting time.

**Methods:**

The development of *Stand Up Australia* followed three phases. 1) Conceptualisation: *Stand Up Australia* was based on social cognitive theory and social ecological model components. These were operationalised via a taxonomy of intervention strategies and designed to target multiple levels of influence including: organisational structures (e.g. via management consultation), the physical work environment (via provision of height-adjustable workstations), and individual employees (e.g. via face-to-face coaching). 2) Formative research: Intervention components were separately tested for their feasibility and acceptability. 3) Pilot studies: *Stand Up Comcare* tested the integrated intervention elements in a controlled pilot study examining efficacy, feasibility and acceptability. *Stand Up UQ* examined the additional value of the organisational- and individual-level components over height-adjustable workstations only in a three-arm controlled trial. In both pilot studies, office workers’ sitting time was measured objectively using activPAL3 devices and the intervention was refined based on qualitative feedback from managers and employees.

**Results:**

Results and feedback from participants and managers involved in the intervention development phases suggest high efficacy, acceptance, and feasibility of all intervention components. The final version of the *Stand Up Australia* intervention includes strategies at the organisational (senior management consultation, representatives consultation workshop, team champions, staff information and brainstorming session with information booklet, and supportive emails from managers to staff), environmental (height-adjustable workstations), and individual level (face-to-face coaching session and telephone support). *Stand Up Australia* is currently being evaluated in the context of a cluster-randomised controlled trial at the Department of Human Services (DHS) in Melbourne, Australia.

**Conclusions:**

*Stand Up Australia* is an evidence-guided and systematically developed workplace intervention targeting reductions in office workers’ sitting time.

## Background

Sedentary behaviour – sitting or lying down while expending little energy [1] - is a newly identified health risk behaviour that is detrimentally associated with several health outcomes, including cardiovascular disease and premature mortality [2-4]. Emerging evidence suggests that both total sitting time and prolonged individual bouts thereof are linked to chronic diseases [5,6]. Further, sedentary behaviour is ubiquitous with adults spending more than half of their waking hours sitting down while watching television, travelling in cars, or working [7,8]. Thus, interventions aiming to reduce sitting time in adults have been identified as an important public health initiative [9].

An opportunistic, high-reach setting for sedentary behaviour intervention is the office-based workplace [10]. Office workers constitute one of the largest occupational groups in industrialised countries such as the US [11], who spend approximately half of their waking hours at work [12]. Importantly, recent studies have shown that they sit for an average of six hours during an eight-hour workday, with this sitting time often accumulated through prolonged unbroken bouts of 30 minutes or more [13-16]. Emerging evidence suggests that targeting workplace sitting through strategies such as modifying the physical work environment [13,17-20], the provision of education sessions and behaviour change advice [16,21,22], or a combination of these strategies [23,24], can be effective. However, information on the development processes of these interventions, such as behaviour change models used and the operationalisation of constructs into intervention messages is limited.

Detailed reporting of intervention development and content is vital to advance intervention research and intervention effectiveness in public health [25]. This should include the theoretical model, targeted context, and formative research and evaluation methods used [10,26-28]. While this type of work is increasingly being published across a number of disciplines including physical activity [29], nutrition [30], chronic disease management [31], and smoking cessation [32] interventions, to the best of our knowledge, no publications have described the development of an intervention to reduce workplace sitting time in adults.

The purpose of this paper is to systematically describe the evidence-based iterative development of the *Stand Up Australia* intervention whose primary aim is to reduce workplace sitting time in office workers. The increasing number of sedentary behaviour publications in both the scientific and popular press over the past decade has led to a demand from office-based workplace settings for assistance with reducing employee sitting. Such requests provided the opportunity for collaborative development of the *Stand Up Australia* intervention, particularly through the formative research phases described below. In accordance with the workplace health promotion literature [33,34] and ecological models of sedentary behaviour [35], *Stand Up Australia* considers the multiple influences on workplace sitting and addresses them via a multi-component approach including behaviour change strategies at the organisational/managerial, environmental, and individual level. The following intervention development description aims to provide a resource for researchers and public health practitioners with a level of detail beyond the restriction of a conventional intervention methods paper.

## Methods

### Identification of an intervention development framework

The systematic development of the *Stand Up Australia* intervention was guided by an intervention development framework. A number of frameworks informed the broader intervention development principles, including the PRECEDE-PROCEED model [36] and the Intervention Mapping approach [37]. Given the specific (workplace) context, a workplace health promotion framework was chosen as the core approach [34], with elements of two other frameworks [38,39] used to complement this method. This included the following key elements: a phased and iterative approach in the development of the intervention [38]; the use of quantitative and qualitative evaluation methods to inform the intervention content [38]; formative research with the target group [39]; and, integration of interrelated dynamics of intra-individual, social, organisational, political, and economic factors within the workplace context [34]. The development of the *Stand Up Australia* intervention involved three phases: 1) Conceptualisation (literature review and theoretical grounding); 2) Formative research (with the target audience); and, 3) Pilot testing of the efficacy, acceptability and feasibility of the integrated multiple components relative to a control group. More specifically, the additional value of the organisational and individual intervention components over an environment (height-adjustable workstations)-only intervention was examined in a three-arm trial. The pilot studies included objective measurement of office workers’ sitting time, as well as quantitative and qualitative data collection from managers and staff.

### Intervention development across 3 phases

#### Phase 1: conceptualisation

*Stand Up Australia* was based on social cognitive theory, which emphasizes the key constructs of self-efficacy, outcome expectancies (physical, social, and self-evaluative), and socio-structural factors (facilitators and impediments) [40]. Evidence on social-cognitive determinants as predictors of sedentary behaviour is still limited [35,41]. However, social cognitive theory has been widely and successfully used in physical activity intervention studies [42]. The operationalisation of theoretical constructs into intervention strategies was guided by an intervention taxonomy [26,43].

Furthermore, *Stand Up Australia* was conceptualised as a multi-component approach to the workplace. Social ecological models of sedentary behaviour emphasize the importance of considering the multiple interrelated influences on individual behaviour. These include the policy environment, the physical and psychosocial environment, and intrapersonal factors [35]. Similarly, best-practice workplace health promotion frameworks identify these influences as key factors for behaviour change strategies in the workplace setting [34,44,45]. In accordance with these models and frameworks, this approach included strategies designed to address organisational structures and the office environment, as well as individuals. By targeting these multiple levels, the aim was to not only raise awareness of sitting behaviours in the workplace, but also to facilitate habitual change via addressing the environment and the workplace culture. Furthermore, key elements in workplace health promotion as identified by the World Health Organization [44] were applied to this sedentary behaviour intervention context as shown in Table 1.

**Table 1:** **Application of “Five Keys to Healthy Workplaces” (World Health Organization) to the *Stand Up Australia *** **Intervention**

**Keys**	**Application to *Stand Up Australia *** **Intervention**
1) Leadership commitment and engagement	- Senior management consultation (gaining leadership commitment, necessary permissions, resources, and support);
	- Representatives consultation workshop (mobilising and gaining commitment from major stakeholders including union representatives and OHS staff)
	- Manager emails (demonstrating continuous management support)
2) Involve workers and their representatives	- Representatives consultation workshop
	- Team champions
	- Staff information and brainstorming session
	- Individual coaching session and telephone support calls
3) Business ethics and legality	- Development of key messages, instructions, and workstation introduction in collaboration with ergonomists and OHS experts
	- Senior management consultation (aligning study principles with workplace policies)
	- Representatives consultation workshop (involvement of OHS staff)
4) Use a systematic, comprehensive process to ensure effectiveness and continual improvement	- Representatives consultation workshop (involving team of multidisciplinary experts)
	- Staff information and brainstorming session (including elaboration of organisational priorities)
	- Pre- and post-intervention assessment of workplace sedentary behaviour in line with key intervention messages
	- Feedback of study results to individuals and the organisation including consultation about future strategies and policy changes
	- Iterative development with continuous improvement of intervention components
5) Sustainability and integration	- Representatives consultation workshop (reducing isolation of work groups, and mobilisation of team champions)
	- Staff information and brainstorming session
	- Assessment of intervention acceptance, feasibility and fidelity
	- Assessment of sedentary behaviour change maintenance
	- Feedback of study results to individuals and the organisation including consultation about future strategies and policy changes

Finally, based on evidence from successful intervention trials on workplace physical activity (the behaviour closest to the one of interest) *Stand Up Australia* is ideally delivered over the course of at least three months [46].

##### Conceptualisation of organisational-level strategies

Effective workplace health promotion interventions address organisational structures and group dynamics through a participative approach and visible management support [33,45]. A participatory approach directly involves staff from all levels (in contrast to a top-down approach) in the identification of well-suited behaviour change strategies and barrier identification. This makes the intervention context-sensitive and appropriate, and thus likely to be implemented and sustained. Within *Stand Up Australia*, this participative approach was implemented through its iterative design including formative research, brainstorming sessions and qualitative feedback interviews. This involved all levels of staff including occupational health and safety (OHS) personnel, workplace safety advisors, and corporate ergonomists (depending on the size of the targeted workplace, this includes senior- and middle managers, as well as team leaders/team champions).

The implementation of *Stand Up Australia* began with initial contact with senior managers within the organisation to elicit support for the study. Further strategies included a representatives consultation workshop and a sedentary behaviour information and brainstorming session for staff, with an accompanying electronic information booklet. During the representatives consultation workshop team champions were selected. They played a crucial part in the identification of behaviour-change opportunities suited to their workplace and in delivering one of the organisational intervention components (sending management emails in support of the study to participating staff).

##### Conceptualisation of environmental-level strategies

Activity-permissive workstations allow office workers to stand, walk, or pedal while working at their usual computer and desk-based job tasks. Examples of activity-permissive workstations include treadmill desks, stepping or pedal devices that are fitted underneath the desk, and height-adjustable workstations. Height-adjustable workstations enable office workers to complete their desk-based and/or computer tasks while alternating between sitting and standing without significant disruption of work practices. Traditionally acquired for the prevention of musculoskeletal problems [47,48], their potential to reduce sitting time for broader preventive-health benefits is increasingly being recognised [10].

Throughout all *Stand Up Australia* study development and implementation phases, manually height-adjustable workstations of the type *WorkFit-S* (manufactured and provided by Ergotron; http://www.ergotron.com) were used. These workstations were chosen as they enabled a ‘retro-fit’ to existing office furniture. They were also less expensive than fully height-adjustable desks. Other environmental-based strategies (e.g. centralisation of printers or in-office waste bins) could be identified in the brainstorming sessions, but the primary focus of this strategy was the use of the height-adjustable workstations.

##### Conceptualisation of individual-level strategies

In line with evidence from successful health intervention programs [49], individual-level intervention strategies were mainly delivered through a face-to-face coaching session with follow-up support telephone calls using a motivational interviewing approach [50]. The face-to-face session followed a script which is very detailed but allowed the consultant to tailor the coaching to the needs of the individual. While there is no firm evidence for an ideal amount of telephone-delivered intervention contact, a recent review suggests that a higher number of telephone contacts is associated with better health behaviour outcomes [51]. In the case of *Stand Up Australia*, where there was an intervention period of three months, four calls were considered to provide an appropriate balance of participant support and time involvement for both participants and researchers.

##### Intervention messages

*Stand Up Australia* targeted three key intervention messages in line with the evidence pertaining to sedentary behaviour and associated health impacts: *Stand Up, Sit Less, Move More. Stand Up* was a prompt to break-up long, unbroken bouts of sitting of 30 minutes or more. This suggestion was based on both epidemiological and laboratory-based evidence which has reported the cardio-metabolic benefits of regularly interrupting sedentary time [5,6]. Furthermore, this target is in line with the ergonomic literature [52,53], and could be practically implemented into office work routines. The message *Sit Less* aimed to reduce total workplace sitting time through substituting some sitting with standing (primarily at the new workstation) and/or moving, with the intent that the reductions in workplace sitting be substantial enough to reduce the health risks associated with high daily sitting time. Finally, the principle of *Move More* was to increase movement throughout the working day. The primary emphasis of this message was on the use of practical strategies (e.g. taking the stairs instead of the lift) to increase incidental physical activity – a key component of daily energy expenditure [54] - throughout the workday.

Table 2 illustrates how these conceptual elements were linked with specific behaviour change strategies related to the key intervention messages of *Stand Up, Sit Less, and Move More* across the three workplace levels (organisational, environmental, individual). This table shows the first iteration of the *Stand Up Australia* intervention. This version of the intervention was used in the formative work with the target audience.

**Table 2:** **Map of *Stand Up, Sit Less, and Move More *** **intervention strategies across intervention target levels**

	**Stand Up**	**Sit Less**	**Move More**
**Principle**	Breaking up prolonged periods of sitting	Reducing overall sitting time	Increasing energy expenditure
**Key message**	Stand up at least every 30 minutes	Reduce daily sitting time	Take every opportunity to be more active
**Organization**	*Focus:*		
	- Changing social norms (reinforcement & role modeling)		
	*Strategies:*		
	- Gain organisational/upper management support through consultation		
	- Identify site representatives as role models and spokespersons for employees		
	- Representatives to reinforce Ix messages (e.g. emails sent from them not research staff, articles in site newsletters)		
	- Establish new workplace policies & practices (e.g. standing meetings, no emails within organisational units- face visits instead, move waste bins, printers, supplies; tailored to each site)		
**Environment**	*Focus:*		
	- Prompts/Behavioral cues	- Use of height-adjustable workstations	- Increasing awareness
	*Strategies:*		
	- Prompts at desk (e.g. postcards, stickers)	- Installation of height-adjustable workstations	- Environmental changes to encourage movement (e.g. signs at lifts prompting use of stairs, centrally located printers & bins; tailored to each site)
	- Timer as visual cue to stand		
**Individual**	*Focus:*		
	- Prompts/Behavioral cues	- Goal setting for use of workstations	- Increasing awareness
	*Strategies:*		
	- Education on breaks in sitting & health	- Education on prolonged sitting & health	- Education on incidental activity & health
	- Encourage use of prompts (e.g. stand when telephone rings, when someone enters the office)	- SMART goal setting for use of workstations	- Encourage use of strategies (e.g. “imails” instead of emails (walk to colleague); walk to bathroom that is farthest away; use stairs instead of lift)
		- Self-monitoring using timer and chart	

#### Phase 2: formative research

The second phase included pilot testing of intervention components at all intervention target levels. This occurred across multiple studies and settings comprising the *Stand Up Australia* program of research.

*At the organisational level,* a consultation session was arranged between senior study investigators and the management of a medium-sized organisation interested in workplace health promotion [24]. This consultation identified this first session as key for gaining management ‘buy-in’, as well as for the identification of organisational processes and structures important to study implementation (e.g. policies around workplace activity).

*At the environmental level,* a preliminary study was conducted testing the efficacy, acceptability, and feasibility of height-adjustable workstations in office workers (Intervention, n = 18; Comparison, n = 14; 94% and 86% women in the intervention and control group, respectively; 20-65 years) between February and June 2011. In this study, and in all other studies forming part of *Stand Up Australia*, evaluation of changes in workplace sitting time was assessed by the activPAL3 activity monitor (PAL Technologies Limited, Glasgow, UK). Detailed methods and results of this preliminary study are published elsewhere [13]. In brief, relative to the comparison group, intervention participants reduced their daily workplace sitting time by an average of 143 minutes per eight-hour day at the workplace following the installation of the workstations (95% CI = -184, -102; p < 0.001), without compromising work-performance. Acceptability of the workstations was high (94% stated that it was enjoyable and easy to use). However, generalisability of these findings was limited due to the intervention sample consisting of a group of public health researchers working in the area of sedentary behaviour research. Furthermore, these findings were limited to closed-plan office designs.

Addressing these limitations, another pilot study was conducted to test the acceptability of the height-adjustable workstations utilised in *Stand Up Australia* in open-plan offices (ethical approval granted by The University of Queensland’s School of Population Health Research Ethics Committee on 4^th^ August 2011; #MN 010811). A convenience sample of five desk-based employees (three women; 20-65 years) was recruited from administrative personnel from a university in Brisbane (Australia) to trial the workstations for two weeks. Following the trial period, all participants (‘workstation group’), as well as another seven employees (‘peer group’; six women; 20-65 years), who shared the same open-plan office and sat nearby the installed workstations, underwent a brief (five-minute) feedback interview on their experience. The interview was semi-structured, audio-recorded, and transcribed.

##### Workstation group feedback

Overall, all five employees were satisfied with the workstations. While suggestions were made for the improvement of the workstation design, everyone appreciated the option to sit or stand while working at their computer – for example, one employee stated: *“It was nice to have the option to sit or stand. It took a lot of pressure off my lower back which usually tends to get sore after prolonged periods of sitting”*. On a 5-point scale (1=’did not like it at all’ to 5=’found it great’), participants rated the workstations from 3 to 5 with an average of 3.9 points. None of the participants perceived any disturbance (visual or auditory) for colleagues working in their immediate environment. Four participants expressed interest in keeping their workstation.

##### Peer group feedback

Six peer group participants did not feel disturbed in any way by others using the workstations. One participant however experienced distraction through the increased noise level and the fact that the ‘workstation user’ was able to look over the partition while standing up - *“We have staff come and see us about confidential/ personal information at our desks. It feels like someone is constantly staring at you*”. Based on the feedback from this participant, a discussion about the purchase of cubicle dividers was taken into the protocol for the management consultation (details below) at the outset of the *Stand Up Australia* Intervention.

*At the individual level,* the feasibility of a face-to-face health coaching session was tested with two university employees (both women, 23 and 28 years) who were not otherwise involved in the *Stand Up Australia* program of research. Overall, the coaching session was received well by the two trial participants and the intended length of the session (30 minutes) was confirmed. Feedback on the key intervention messages led to further clarification of the distinction between *Stand Up* (i.e. standing up regularly to break up long bouts of sitting) and *Sit Less* (i.e. reducing the overall sitting time throughout the day by replacing some sitting time with standing and/or moving time).

#### Phase 3: pilot testing

Two pilot studies were conducted and are described below: ‘*Stand Up Comcare’*, a two-arm controlled trial that tested the efficacy, feasibility, and acceptability of the integrated multiple components; and, *‘Stand Up UQ’*, a three-arm controlled trial that evaluated the additional value of the multiple components over height-adjustable workstations only.

##### *Stand Up Comcare* methods

An abridged, four-week version of the *Stand Up Australia* intervention was initially pilot tested with 43 employees (56% women; 26-62 years) in a two-arm controlled trial between July and September 2011 in an urban open-plan office (*Comcare*: the government agency responsible for workplace safety, rehabilitation and compensation for Australian government workplaces) in Melbourne, Australia [24]. The main purpose of this pilot was to test the combined implementation of all three intervention components. Following the pilot study, intervention group participants completed a telephone interview about their study experience and managers provided feedback in face-to-face sessions.

##### *Stand Up Comcare* results

Results from this pilot study are published elsewhere [24]. In brief, relative to the control group, participants in the intervention group reduced their workplace sitting time by just over two hours per eight-hour workday (mean change -125, 95% CI = -161, -89 minutes) following intervention, with sitting primarily replaced with standing (127, 95% CI = 92, 162 minutes). Of the 21 intervention group participants, 18 completed the telephone feedback interview. Overall, the height-adjustable workstations, as well as the organisational and individual intervention components (in particular the face-to-face coaching session), were evaluated very positively by both staff and managers (detailed below).

##### Intervention refinement based on *Stand Up Comcare*

Based on the feedback from *Stand Up Comcare*, the *Stand Up Australia* intervention was modified at all three levels. Regarding *organisational-level* strategies, the majority of participants indicated that the initially standardised manager emails were mostly left unread due to email overload and not enough relevance – for example, *“I read one or two but don’t remember more than that, didn’t take much notice of them”.* Thus, the intervention was refined to tailor the manager email templates provided by the study to the managers’ observations of their team’s experience with the intervention. This could include the observation of potential problems (e.g. sore feet from increased levels of standing) and suggested solutions (e.g. keeping a spare pair of orthopaedic shoes at the desk). While the primary outcome targeted through *Stand Up Australia* is sitting at the workplace, it was decided to add a list of useful strategies to *Stand Up, Sit Less, and Move More* outside of the workplace to the second manager email. Further, during the management consultation, more emphasis was placed on the initiation of standing by managers/senior-level staff during staff meetings, as participants repeatedly expressed feeling ‘awkward’ to initiate standing by themselves - *“I find it hard to stand in a meeting when no one else is doing it - uncomfortable”*. Finally, the list of organisational strategies to promote standing proposed to managers was refined based on feedback on the most and least useful strategies identified by both staff and managers. *At the environmental level*, a detailed ergonomic introduction to the height-adjustable workstations (involving internal OHS staff wherever available) was added to the intervention protocol. This introduction was delivered immediately following the workstation installation to address employee concerns about their limited experience in the correct use of these. During the *individual intervention contacts*, a stronger emphasis was placed on the importance of regular postural changes, and, as most participants experienced difficulties distinguishing the principles of *Stand Up* and *Sit Less,* a clearer and more detailed explanation of these recommendations was incorporated. Further, assisting participants with the set-up of a stopwatch or computer software to monitor their sitting/standing time if required was included in the protocol, as some participants experienced difficulties with doing this on their own. In line with the addition of a strategy list to *Stand Up, Sit Less,* and to give more emphasis to the target of *Move More* outside of the workplace, a discussion about these strategies was added to the protocol of the third telephone call in addition to the related list of strategies added to the second manager email. Finally, the email summaries sent following the telephone calls were removed from the intervention protocol, as feedback from employees indicated that these were generally not read due to an overload of emails.

The results of this pilot study addressed the efficacy considerations of the multi-component intervention on reducing workplace sitting time. However, as the participating employees were from a government agency for workplace safety, rehabilitation and compensation, the results may be limited in their generalizability. Furthermore, based on the two-group design it was not possible to determine the contribution of the organisational- and individual-level elements, as distinct from the provision of height-adjustable workstations alone. Considering the resource implications of these elements, this issue has important practical and financial implications. The second pilot study (*Stand Up UQ*) therefore involved a test of the efficacy of this multi-component intervention to a height-adjustable workstations-only intervention in a three-arm controlled trial involving a comparatively representative sample of office workers.

##### *Stand Up UQ* methods

Between January and June 2012, a group of desk-based office workers from three separate administrative units of The University of Queensland (Brisbane, Australia) participated in the ‘*Stand Up UQ*’ study (multi-component intervention, n = 16; height-adjustable workstations-only, n = 14; comparison, n = 14; 84% women; 20-65 years). The multi-component intervention comprised all the *Stand Up Australia* intervention elements as refined following the *Stand Up Comcare* pilot study (detailed above), delivered over three months. Participants in the workstations-only intervention received height-adjustable workstations only.

##### *Stand Up UQ* results

Results are published elsewhere [55]. In brief, following intervention and relative to the comparison group, workplace sitting time in the multi-component group was reduced by 89 mins/8-hour workday (95% CI = -130, -47 minutes; p < 0.001) and 33 minutes in the workstations-only group (95% CI = -74, 7 minutes, p = 0.285). Furthermore, all participants in the multi-component intervention rated all intervention components as either useful or very useful. In particular, 12/13 rated the manager emails, which were mostly left unread in the *Stand Up Comcare* pilot and therefore tailored in the refinement, as either useful or very useful (one participant was neutral) – *“Her emails brought everyone onto the same page and encouraged [us] to try things, reinforcing support”.*

## Results (final intervention design)

The following section provides a detailed description of the resulting *Stand Up Australia* intervention protocol. The suggested timing of all intervention components is shown in Table 3.

**Table 3:** Intervention elements and timing of implementation

	***Timing***	**Intervention level**		
		**Organisational**	**Environmental**	**Individual**
**Intervention elements**	*Week 1*	Senior management consultation		
	*Week 2*	Representatives consultation workshop		
	*Week 3*	Staff information & brainstorming session; Manager email 1	Workstation installation	Coaching session & email summary
	*Week 4*			Phone call 1
	*Week 5*	Manager email 3		
	*Week 6*			Phone call 2
	*Week 7*	Manager email 4		
	*Week 8*			
	*Week 9*	Manager email 5		Phone call 3
	*Week 10*			
	*Week 11*	Manager email 6		
	*Week 12*			
	*Week 13*	Manager email 7		Phone call 4

### Organisational intervention strategies

In brief, there are three key strategies targeting the organisational level: A senior management consultation, a representatives consultation workshop, and a staff information and brainstorming session including the provision of an information booklet.

#### Senior management consultation (approx. 30-45 mins)

During a consultation session between senior research staff (trained in the evidence of excessive sitting and detrimental health outcomes) and selected senior staff, details of the study timeline are presented and an explanation of the role of organisational and physical environmental factors in determining occupational sitting time is given. Furthermore, current organisational processes and structures important to study implementation are considered, the concept of the representatives consultation workshop is introduced, and relevant staff identified (more details below). Strategies to encourage employee participation are discussed and important OHS policies and resources identified (e.g. those relating to workplace activity). Finally, additional resources to support study targets are identified (e.g. headphones or higher partitions between desks).

#### Representatives consultation workshop (approx. 2-4 hours)

Staff representatives meet with senior research staff to identify strategies supportive of behaviour change (in line with *Stand Up Australia* key intervention messages) suitable to their organisation. Representatives ideally include staff from each staff level (including senior and middle managers) as well as other important stakeholders such as OHS personnel, union representatives, workplace safety advisors, and corporate ergonomists. During the workshop, research staff present details on the research background and target behaviour, and representatives identify feasible workplace changes to *Stand Up, Sit Less, and Move More* suited to their organisation (e.g. standing meetings, or the relocation of printers and waste bins). Furthermore, a group of team champions is identified. Throughout the duration of the *Stand Up Australia* intervention, the role of team champions is: 1) to actively promote standing by using their height-adjustable desks and to encourage and initiate standing in staff meetings (e.g. by hanging up signs in meeting rooms that ‘standing meetings are welcome’ or by announcing in the beginning of a staff meeting that staff are welcome to stand); 2) to act as liaison between staff and the research team; and, 3) to distribute the management emails (one champion; typically a manager). Standard email templates supportive of the study targets are provided to this champion by the research team. The champion is asked to walk through the offices on a regular basis to observe and chat to staff about potential problems related to the new workstations or other study components. Any observations are subsequently integrated into the email templates. Six fortnightly emails are sent to staff (blind copied to the research team) over the course of the three-month intervention.

#### Staff information and brainstorming session and information booklet (approx. 30-45 mins)

Research staff facilitate a staff information and brainstorming session. This session addresses the detrimental health impacts of prolonged sitting and provides details about intervention participation, as well as feedback on the group’s workplace sitting time collected from the activity monitors at baseline. Organisational strategies to *Stand Up, Sit Less, and Move More* as identified in the representatives consultation workshop are discussed, and staff are encouraged to further brainstorm strategies that may be specifically suited to their group. Following this information session, a summary email (provided by research staff) is sent from the responsible team champion to all staff. This email includes an electronic *Stand Up Australia* information booklet with details about: the study rationale (i.e. evidence on sedentary behaviour and health outcomes) and purpose; general guidelines on optimal workplace activity; specific behaviour change strategies related to the key intervention messages; and, general information about the study procedure and timeline.

### Environmental intervention strategy

#### Height-adjustable workstation installation and ergonomic posture check

Each participating employee receives a height-adjustable workstation. In consultation with an OHS ergonomist, the authors adapted the manufacturer-provided workstation information sheet, which contains details about the correct ergonomic posture and tips on the use of the workstation, as well as the study recommendations on workplace sitting and activity. It is left on the workstation shelf for each participant to read upon the first contact with the new workstation. Following the workstation installation, the organisation’s OHS staff confirm the correct ergonomic posture and address any workstation-related problems or questions. If OHS staff are unavailable, this step is conducted by research staff following study-specific training.

### Individual intervention strategies

In brief, the individual component consists of one face-to-face coaching session and four follow-up support telephone calls over the three months.

#### Face-to-face coaching

The workstation installation is followed by an individual 30-minute face-to-face coaching session delivered by a health coach (trained in motivational interviewing techniques) in a private room at the work site. First, the participant and coach review the participant’s individual feedback document [55]. This analytic feedback document reflects the participant’s activity and posture (as recorded during the baseline assessment week) in relation to each of the key messages *Stand Up, Sit Less, and Move More*. This includes both overall proportions of sitting, standing, and moving time across the day and during work hours only, as well as a 24 hour ‘heatmap’ showing times at which these activities occurred for each day during the assessment week. Then, potential disparities between the baseline and target behaviours are established, and specific goals for each key message are elaborated on using motivational interviewing methods. For example, tasks undertaken during long periods of sitting visible on the heat map are discussed and solutions to achieve the desired behaviour target identified. Goals are documented on a ‘Workstation Tracker’ [55], which is to be attached to the workstation clearly visible to the participant (for self-monitoring purposes). Following the coaching session, participants receive an email summary from the coach containing the key points discussed.

#### Support telephone calls

Each intervention group participant receives a total of four behaviour change support telephone calls following the coaching session, preferably from the same health coach. The telephone calls are delivered in staggered intervals (preferably at one, three, six, and ten weeks following the coaching session), offering more intense support during the initiation period and gradually less during the maintenance period of behaviour change. They serve as a general check-in on the participants’ satisfaction with the study and their workstation, their goal achievement, barrier identification and problem solving, discussion of new strategies, and a potential adjustment of goals. During the second call, the health coach also discusses strategies to *Stand Up, Sit Less, and Move More* outside the workplace. On average, these calls should take around ten minutes.

#### Intervention feedback

At the end of the three-month intervention period, and again one year after baseline, the research team provides both individuals and the organisation with feedback on the sitting time reductions experienced by staff. This can be accompanied by a consultation on strategies that were considered to be most suited to the particular organisation and a discussion regarding potentially relevant future strategies and policy changes.

## Discussion

Detailed reporting on intervention development is vital for the advancement of effective behaviour change interventions. This is the first paper to provide a thorough description of the development process of an intervention to reduce sitting time in office workers - *Stand Up Australia*.

Key strengths of this development process include: a systematic three-stage process guided by currently available evidence; strong theoretical grounding and translation of key constructs guided by the use of an intervention taxonomy; a participative approach to both the broader workplace and its staff; the targeting of multiple levels of influence on workplace sitting (organisational/managerial, environmental, and individual); as well as the integration of qualitative and quantitative data to inform subsequent uptake into practice.

However, when considering the potential for wide-spread translation of the *Stand Up Australia* intervention, it should be noted that, despite the strong input from workplaces into intervention development and the pragmatic design of the evaluation, the participatory process was limited by research funding constraints. The findings may therefore not generalise across the wider population of office workplace settings.

*Stand Up Australia* is currently being evaluated in the context of a cluster-randomised controlled trial at the Department of Human Services (DHS) in Melbourne, Australia (*Stand Up Victoria*; ACTRN12611000742976). This study is funded by the Australian National Health and Medical Research Council and the Victorian Health Promotion Foundation, and includes objective measurement of activity and posture via activPAL3 monitors, clinical assessment of anthropometric outcomes and cardio metabolic biomarkers, evaluation of work-related outcomes (including productivity, absenteeism and presenteeism), as well as cost-effectiveness analyses [56].

## Conclusions

*Stand Up Australia* is an evidence-informed and systematically developed workplace intervention targeting reductions in office workers’ sitting time. Feedback from participants and managers involved in the multiple phases of development suggests high acceptance and feasibility of all intervention components. Observations from the pilot studies demonstrate the efficacy of *Stand Up Australia* to significantly reduce office workers’ sitting time. Results of the currently implemented cluster-randomised controlled trial will inform its (cost-) effectiveness and feasibility on a larger scale.

## Consent

Written informed consent was obtained from all participants for the publication of this report and any accompanying images.

## Competing interests

Height-adjustable workstations were kindly provided by Ergotron (http://www.ergotron.com) for the *Stand Up Comcare* and *Stand Up UQ* pilot studies. The funding bodies had no influence on the conduct or the findings of the study.

## Authors’ contributions

All authors made substantial contributions to the conceptualisation and development of the *Stand Up Australia* intervention. All authors played a significant role in the drafting of this manuscript. All authors read and approved the final manuscript.
